# Gastroenteropancreatic Neuroendocrine Tumor with Peritoneal Metastasis: A Review of Current Management

**DOI:** 10.3390/cancers16203472

**Published:** 2024-10-14

**Authors:** Corey A. Hounschell, Simon Higginbotham, Mazin Al-Kasspooles, Luke V. Selby

**Affiliations:** 1 Department of Surgery, University of Kansas Medical Center, Kansas City, KS 66103, USA; chounschell@kumc.edu (C.A.H.); mal-kasspooles@kumc.edu (M.A.-K.); 2 University of Kansas School of Medicine, Kansas City, KS 66160, USA; shigginbotham@kumc.edu

**Keywords:** neuroendocrine tumor, peritoneal metastasis, cytoreductive surgery, HIPEC, endocrine therapy, targeted drug therapy

## Abstract

**Simple Summary:**

Many patients diagnosed with gastroenteropancreatic neuroendocrine tumors present with metastatic disease at the time of diagnosis. As many as 20% of patients with this diagnosis will present with metastasis to the peritoneal cavity which portends a significantly worse prognosis than solid organ metastasis alone. There is little to no evidence to guide treatment algorithms given the rarity of this disease. Surgical cytoreduction has been found to improve symptoms related to disease burden, though an understanding of the optimal cytoreductive strategies to offer the greatest benefit to most patients is lacking. While the current body of literature pertaining to the systemic treatment of the solid organ metastasis of this disease is more robust, it largely excludes patients with peritoneal metastasis. We provide a review of the current literature which is used to guide these management strategies and offer insight into the knowledge gaps that exist.

**Abstract:**

Peritoneal metastasis in gastroenteropancreatic neuroendocrine tumors poses a significant clinical challenge, with limited data guiding management strategies. We review the existing literature on surgical and systemic treatment modalities for peritoneal metastasis from gastroenteropancreatic neuroendocrine tumors. Surgical interventions, including cytoreductive surgery, have shown promise in improving symptom control and overall survival—particularly in cases in which 70% cytoreduction can be achieved. Hyperthermic intraperitoneal chemotherapy remains controversial due to a paucity of high-level evidence and a lack of consensus for routine use. The use of systemic therapy in the setting of peritoneal metastasis from gastroenteropancreatic neuroendocrine tumors is extrapolated from high-quality evidence for its use in the setting of the solid organ metastasis of this disease. The use of somatostatin analogs for symptom control and some antiproliferative effects is supported by large clinical trials. Additional strong evidence exists for the use of interferon-alpha, everolimus, and sunitinib, particularly in pancreatic neuroendocrine tumors. Cytotoxic chemotherapy and peptide receptor radionuclide therapy may be used in select cases, though as an emerging treatment modality, the optimal sequence of peptide receptor radionuclide therapy within the existing algorithms is unknown. Significant gaps in understanding and standardized management exist, particularly for those patients presenting with peritoneal metastasis, and targeted research to optimize outcomes in this population is needed.

## 1. Introduction

Approximately 20% of the patients diagnosed with gastroenteropancreatic neuroendocrine tumors present with peritoneal metastasis. Often, other sites of lymphatic and solid organ metastasis are concurrently present in these patients, with the liver being the most common site of metastatic disease, present in 71% of patients in one series [1,2]. Depending on the origin of the primary tumor, metastatic disease may be associated with a 5-year survival of <50%. The presence of hepatic metastasis and tumor grade has been identified as the most important prognostic factors associated with metastatic gastroenteropancreatic neuroendocrine tumors; however, the presence of peritoneal metastasis has been shown by some small studies to adversely affect survival when compared to patients with solid organ metastasis only [2,3,4,5,6]. Given the rarity of gastroenteropancreatic neuroendocrine tumors with peritoneal metastasis, there is limited data driving its management. Most recommendations are derived from the extrapolation of data related to solid organ metastasis. The following is a summary of the current treatment strategies in the management of peritoneal metastasis from gastroenteropancreatic neuroendocrine tumors.

## 2. Surgical Management

### 2.1. Cytoreductive Surgery (CRS)

Multiple retrospective studies have evaluated the role of cytoreduction in the setting of metastatic gastroenteropancreatic neuroendocrine tumors for both the palliation of symptoms and improvement of overall survival. A study published by McEntee et al. [7] was one of the first to evaluate the role of cytoreduction in metastatic gastroenteropancreatic neuroendocrine tumors and improvement in the symptoms related to the disease. Thirty-seven patients undergoing hepatic resection for metastatic gastroenteropancreatic neuroendocrine tumors between 1970 and 1989 were reviewed by the authors. Of the patients studied, 17 underwent resection with curative intent, 9 underwent resection for symptomatic endocrinopathies, and 7 underwent resection to palliate symptoms caused by the mass effect of the primary tumor. The authors reported good symptom control with cytoreduction though patient-reported outcome measures were not reported. Based on their institutional experience, the authors recommended the consideration of cytoreductive surgery if a >90% cytoreduction could be achieved. Though this 90% cytoreductive threshold was without clear justification, it was subsequently adopted as the gold standard approach to cytoreductive surgery in this setting and was endorsed by many future authors who used this threshold to offer intervention [7].

Several subsequent studies were published with attention focused on not only the improvement of symptomatology related to metastatic burden, but also improvement in the overall survival associated with cytoreduction. Chambers et al. [8] evaluated the efficacy of aggressive surgical intervention in the palliative setting. A review of 66 patients with metastatic gastroenteropancreatic neuroendocrine tumors was performed. The patients were managed with a standardized protocol of the initial surgical resection of regional and/or hepatic disease and subsequent medical therapy. Of the patients included, 36% presented with symptoms of obstruction or ischemia and 85% presented with carcinoid syndrome. Of patients with obstructive symptoms, 100% experienced complete symptomatic relief after the surgical intervention. Overall, 75% of the patients presenting with carcinoid syndrome experienced improvement in symptoms after cytoreduction. Thirty patients underwent hepatic resection, and of those, 86% experienced improvement in carcinoid symptoms compared to 64% of the patients who did not [8].

Woltering and colleagues [9] were the first to publish a large series of patients undergoing cytoreduction for metastatic gastroenteropancreatic neuroendocrine tumors of multiple primary tumor locations. Of the 800 patients included, 65% had small bowel primary neuroendocrine tumors, 11% had neuroendocrine tumors of pancreatic origin, and smaller numbers presented with neuroendocrine tumors of the lung, gastric, appendiceal, colonic, rectal, and unknown primary location. The overall survival and actuarial survival were reported, with actuarial survival being defined as the life expectancy of a similar patient cohort without a neuroendocrine tumor diagnosis. The 5-, 10-, and 20-year overall survival were reported to be 82%, 65%, and 37%, respectively, with a median improvement in life expectancy after cytoreductive surgery of 11.2 years (difference in median actuarial survival and median overall survival). A subset analysis was performed to evaluate survival benefits relative to the degree of cytoreduction performed. The two largest disease sites studied were included in this subset analysis (pancreatic and small bowel primary). In patients with metastatic pancreatic neuroendocrine tumors, the authors reported a difference in median overall survival of only 5 months in those patients undergoing a cytoreduction of 90–98% (group 1) and those undergoing a cytoreduction of <90% (group 2) of their disease. The 5-, 10-, and 20-year overall survival was 68%, 41%, and 41%, respectively, for group 1, and 56%, 28%, and 40%, respectively, for group 2. In patients with small bowel primary neuroendocrine tumors undergoing cytoreductive surgery, the authors reported similar results but also reported outcomes for those undergoing a cytoreduction of 70–89% and <70% of their disease burden. Five-, ten- and twenty-year overall survival for those undergoing 70–89% cytoreduction in this cohort were reported to be 89%, 64%, and 25%, respectively, compared to 64%, 40%, and 13%, respectively, for those undergoing a cytoreduction of <70%. While increasing degrees of cytoreduction were shown to be significantly associated with an increase in overall survival, the authors argued that a cytoreductive threshold of 70% should be adopted as standard given the reasonable survival outcomes, especially when compared to medical management or palliation alone [9]. This argument is supported by the findings of Maxwell et al. [10] who demonstrated improvement in progression-free survival and overall survival in patients with small bowel and pancreatic neuroendocrine tumors undergoing a cytoreduction of >70% [10]. This 70% threshold has since been adopted as the gold standard.

Though lowering the threshold for offering cytoreduction from a 90% reduction in tumor burden to 70% greatly increases the number of patients who would qualify for this approach, there remain many patients in whom even this degree of reduction is not possible. In this setting, though controversy remains, some studies have demonstrated improved survival for patients with unresectable disease undergoing the resection of primary tumor only.

Tierney et al. [11] evaluated 14,510 patients from the National Cancer Database with metastatic gastroenteropancreatic neuroendocrine tumors undergoing the resection of primary tumor only. The authors reported an increase in overall survival with primary tumor resection compared to non-operative management for the neuroendocrine tumors of primary pancreatic, small intestinal, colonic, rectal, and gastric origin. An increase in the overall survival of 49.4 months with primary site resection compared to non-operative management was seen in the pancreatic group. Patients diagnosed with grade 1 or grade 2 tumors and those younger than the median age of 57 years experienced the greatest survival benefit. Of those with neuroendocrine tumors of small bowel primary, an increased length of survival by 47.1 months was observed in the same context. Patients with grade 1–2 tumors and younger than the median age (64 years) experienced the greatest benefit. Similar survival benefits were seen with colorectal and gastric primary tumors, though to a lesser extent. The resection of colonic primary yielded a benefit of 8.1 months, rectal 12.5 months, and gastric 14.2 months. All showed the greatest survival benefit in patients with grade 1–2 tumors [11].

A similar study published by Lewis and colleagues [12] demonstrated similar results. A total of 854 patients with metastatic gastroenteropancreatic neuroendocrine tumors were included from the California Cancer Registry and California Office of Statewide Health Planning and Development database. These authors reported an improvement in the 5-year overall survival of 35.8% for primary pancreatic neuroendocrine tumors, 36% for small bowel primary, and 15.2% for colorectal primary. In contrast to the study by Tierney et al., the patients included in this analysis were allowed to undergo concurrent treatment of liver metastasis. Notably, the concurrent treatment of liver metastasis and resection of the primary tumor only offered a significant increase in the 5-year overall survival in those patients with pancreatic neuroendocrine tumors (56.8% vs. 48.9%) [12].

### 2.2. Hyperthermic Intraperitoneal Chemotherapy (HIPEC)

Many randomized and prospective studies examine the use of hyperthermic intraperitoneal chemotherapy in the setting of various intra-abdominal malignancies with conflicting results regarding any benefit to disease-free and overall survival [13,14,15,16]. Unfortunately, no such randomized controlled trials exist or are in the process of evaluating the use of hyperthermic intraperitoneal chemotherapy for the treatment of gastroenteropancreatic neuroendocrine tumors with peritoneal metastasis. So, while the benefit of cytoreduction in this setting is well demonstrated, the role of hyperthermic intraperitoneal chemotherapy is less understood given that only a few small retrospective studies and case series exist which explore its use.

A systematic review published in 2023 by Fallows et al. [17] identified only four studies in which hyperthermic intraperitoneal chemotherapy was used in conjunction with surgical cytoreduction in the setting of metastatic gastroenteropancreatic neuroendocrine tumor (Table 1). The earliest, by Elias et al., [18] included 41 patients. Twenty-eight patients underwent cytoreduction and hyperthermic intraperitoneal chemotherapy while thirteen patients underwent hyperthermic intraperitoneal chemotherapy alone. The patients in this study were included only if CC-0 resection was performed (based on the Sugarbaker Completeness of Cytoreduction scoring system). Those who underwent hyperthermic intraperitoneal chemotherapy received an intravenous infusion of 20 mg/m^2^ leucovorin followed by 400 mg/m^2^ 5-fluorouracil. The intravenous administration of chemotherapy was followed by a 30 min administration of oxaliplatin alone (460 mg/m^2^) or oxaliplatin and irinotecan combined (300 mg/m^2^ and 200 mg/m^2^, respectively). The authors did not indicate the number of patients who received the hyperthermic intraperitoneal chemotherapy regimen. The intraperitoneal chemotherapy was administered at 43 °C. Comparative overall survival and disease-free survival were reported at 1 and 2 years. One- and two-year disease-free survival was reported to be superior in the hyperthermic intraperitoneal chemotherapy group at 77% and 49%, respectively, compared to 49% and 16% (*p* = 0.018), though the overall survival during this period between the groups did not differ [18].

Brandl et al. [19] published a 14-patient case series of patients undergoing cytoreductive surgery and heated intraperitoneal chemotherapy for “rare diseases”. Only one patient underwent the procedure for metastatic neuroendocrine tumors of gastric origin. This patient had disease recurrence after 13.5 months and was alive at the time of publication (48.9 months from the date of surgery). The authors in this study did not disclose the intraperitoneal chemotherapy agent used or the time and temperature of treatment administered [19]. Goere and colleagues [20] published a similar case series. All the institutions associated with the Peritoneal Surface Oncology Group International were surveyed regarding their use of heated intraperitoneal chemotherapy in rare peritoneal malignancies. Of the patients included in the case series, 127 underwent cytoreductive surgery and hyperthermic intraperitoneal chemotherapy for neuroendocrine tumors. Specifics of the intraperitoneal chemotherapy regimen used for these patients were not explicitly described. The five-year overall survival for those patients with gastroenteropancreatic neuroendocrine tumors who underwent cytoreductive surgery and hyperthermic intraperitoneal chemotherapy was reported at 39.9% and the five-year disease-free survival reported as 40.2%. The patients who did not achieve at least CC-1 cytoreduction were excluded from the disease-free survival analysis. No comparison to the patients undergoing cytoreductive surgery alone was reported in this series [20].

A more recent retrospective review by Hajjar et al. [21] reviewed patients from the PSOGI and BIG-RENAPE working group databases who underwent cytoreductive surgery alone or in conjunction with hyperthermic intraperitoneal chemotherapy for metastatic small bowel neuroendocrine tumors with peritoneal metastasis. Sixty-seven patients were included with thirty-six undergoing cytoreduction and hyperthermic intraperitoneal chemotherapy and thirty-one undergoing cytoreduction alone. Details on the specific intraperitoneal chemotherapy regimens used were not reported. Morbidity data were reported with 50% of the patients undergoing hyperthermic intraperitoneal chemotherapy experiencing Clavien–Dindo grade III-IV complications versus 3.4% of those undergoing cytoreduction alone. There was no statistically significant difference in the overall survival, progression-free survival, or recurrence-free survival at 5 years between those patients undergoing cytoreduction and the administration of intraperitoneal chemotherapy versus cytoreduction alone [21].

**Figure 1:**
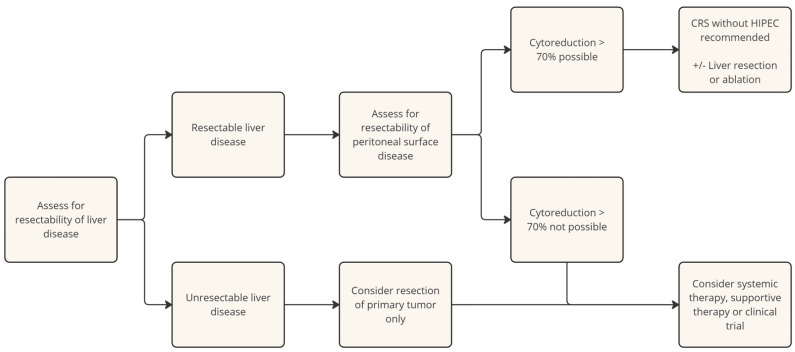
Treatment algorithm for gastroenteropancreatic neuroendocrine tumors with peritoneal metastasis proposed by the Chicago Consensus Working Group for Peritoneal Surface Malignancies [22].

The Chicago Consensus Working Group published a multidisciplinary consensus statement regarding the management of the peritoneal metastasis of neuroendocrine tumor [22]. This statement recommends the cytoreduction of at least 70% of the tumor burden. If this degree of cytoreduction is not possible, they support the consideration of isolated primary tumor resection due to its possible benefit on survival, though acknowledge that evidence is lacking. Given the paucity of high-level evidence in support of the use of hyperthermic intraperitoneal chemotherapy in conjunction with cytoreduction, its use is not recommended by the Chicago Consensus Working Group. Systemic therapy is recommended in the case that cytoreduction is not possible or desired. The treatment algorithm below is derived from the recommendations of this consensus statement (Figure 1) [22].

## 3. Systemic Therapy

Most systemic therapies aimed at the treatment of metastatic gastroenteropancreatic neuroendocrine tumors have limited efficacy, including more historic streptozocin-based chemotherapy regimens which have largely fallen out of favor [23,24]. Given the paucity of data regarding the efficacy of these medications in the setting of peritoneal metastasis, their use in this context is even less well understood. Management is, therefore, guided by the extrapolation of data from studies examining patients with solid organ metastatic disease [1,25]. The following is a review of the currently utilized systemic treatment approaches for metastatic gastroenteropancreatic neuroendocrine tumors and their supporting evidence as it applies to the management of peritoneal metastasis.

### 3.1. Somatostatin Analogs

The somatostatin analogs, octreotide and lanreotide, are considered first-line therapy for patients with functional gastroenteropancreatic neuroendocrine tumors and the associated carcinoid syndrome as a means of symptom control. These agents have been found to have equal efficacy in this role and can be used safely as long-term therapy [26]. Though generally effective, some patients experience carcinoid syndrome refractory to the use of long-acting octreotide or lanreotide. One phase II trial demonstrated symptom improvement in 27% of patients with carcinoid syndrome refractory to the standard octreotide dosages with the administration of pasireotide [27]—a novel universal somatostatin ligand approved for the treatment of functional pituitary tumors—however, a later trial demonstrated no superiority compared to high doses of long-acting octreotide and therefore, its use has not been widely adopted in this setting [28].

In addition to the control of the symptoms related to carcinoid syndrome, somatostatin analogs have demonstrated efficacy as antiproliferative agents against intestinal neuroendocrine tumors, and consensus among experts is that somatostatin analogs should be initiated at diagnosis in the case of advanced metastatic disease. The PROMID [29] and CLARINET [30] studies are two placebo-controlled trials that demonstrated the good antiproliferative action of both octreotide and lanreotide in the treatment of gastroenteropancreatic neuroendocrine tumors. PROMID examined treatment-naive patients with well-differentiated metastatic midgut neuroendocrine tumors. Patients received long-acting octreotide or placebo with the primary endpoint examined being time to tumor progression and secondary endpoints being survival time and tumor response. The patients receiving octreotide in this study population exhibited a median progression-free survival of 14.3 months compared to 6 months in the placebo group. The authors noted that functional and non-functional tumors responded similarly to the treatment and that the patients with a low hepatic tumor burden and previously resected primary tumor responded most favorably [29].

The CLARINET trial examined patients with well- to moderately differentiated, nonfunctioning, somatostatin receptor-positive gastroenteropancreatic neuroendocrine tumors. The primary endpoint of this trial was progression-free survival or death, and the secondary endpoints included overall survival and quality of life. Patients receiving lanreotide demonstrated significantly better outcomes with 65.1% progression-free survival at 24 months compared to 33% in the placebo group. No difference in the quality of life or overall survival was observed between the groups [30]. Though the antiproliferative properties of somatostatin analogs were established as a class effect by the CLARINET study, the PROMID study excluded those patients with pancreatic neuroendocrine tumors, and therefore, lanreotide is the only somatostatin analog supported by prospective data for use in this population. Though some retrospective data exist to support the use of octreotide in low-grade pancreatic neuroendocrine tumors, lanreotide is still preferentially used in this population [31].

### 3.2. Interferon-Alpha

Interferon-Alpha, a cytokine that mediates antiproliferative activities, may in some cases be used as second-line therapy in addition to somatostatin analogs for symptom control in the case of refractory carcinoid syndrome or in patients with advanced neuroendocrine tumor. One small randomized clinical trial examined the effect of interferon-alpha on patients with metastatic midgut neuroendocrine tumors. Sixty-eight patients who had undergone primary resection and the hepatic arterial embolization of liver metastasis were randomized to treatment with octreotide alone or a combination of octreotide/interferon-alpha. Though no difference in overall survival was identified between the two arms, the patients treated with combination therapy were found to have a significantly lower risk of disease progression (HR 0.28) [32]. The SWOG S0518 trial compared progression-free survival in 402 patients with unresectable or metastatic grade 1/2 neuroendocrine tumors demonstrating features of poor prognosis who received octreotide plus bevacizumab or octreotide plus interferon-alpha. The primary outcomes of this study included progression-free survival or death and the secondary outcomes included site-reported progression-free survival, overall survival, time to treatment failure, objective response, and drug toxicity. The results demonstrated no difference in the progression-free survival or overall survival between the two study arms [33].

### 3.3. Everolimus and Sunitinib

Everolimus and sunitinib are both approved first-line therapies for progressive pancreatic neuroendocrine tumors. One study demonstrated improved progression-free survival (11.4 months versus 5.5), overall survival, and objective response to therapy in patients with metastatic well-differentiated pancreatic neuroendocrine tumors receiving sunitinib compared to placebo [34]. The RADIANT-3 trial examined and reported similar results with the use of everolimus in a similar patient population. In this study, the median progression-free survival was 11 months in the study group compared to 4.6 months in the placebo group [35].

Though both agents have demonstrated efficacy in the treatment of pancreatic neuroendocrine tumors, only everolimus has been shown to improve progression-free survival in neuroendocrine tumors of non-pancreatic origin. The RADIANT-4 trial demonstrated a 52% reduction in the estimated risk of progression or death in the patients receiving everolimus compared to placebo [3]. Everolimus is often used as cross-over therapy in conjunction with streptozotocin-5FU chemotherapy regimens. The recently completed SEQTOR study compared the rate of progression-free survival in patients with grade 1/2 pancreatic neuroendocrine tumors who received this strategy of sequential therapy and aimed to establish the most effective order of treatment. Though the overall rates of progression-free survival were similar between the two arms, the patients who received everolimus as the second agent in the order of treatment demonstrated a greater objective response to the treatment [36]. Toxicities in the face of other comorbid conditions may drive patient selection in the use of everolimus in this setting.

Though it is standard practice to use targeted drug therapies in conjunction with somatostatin analogs in the case of functionally active, metastatic neuroendocrine tumors, there is no strong data supporting combination therapy in non-functional tumors [37,38].

### 3.4. Cytotoxic Chemotherapy

Cytotoxic chemotherapy finds its most frequent use in patients with grade 1/2 pancreatic neuroendocrine tumor, bulky disease, symptoms related to tumor burden, rapid disease progression, or who are borderline surgical candidates [39,40,41]. The most accepted drug combinations include streptozotocin/5-FU and doxorubicin/streptozotocin with a variety of regimens described. Though the quality of supporting evidence is much lower, tomozolomide/capecitabine is used as an alternative regimen to streptozotocin/5-FU in some cases or as alternative therapy after the failure of streptozotocin-based regimens. The use of this alternative regimen is supported by small sets of prospective and retrospective data with a wide range of rates of objective response reported [42,43].

In general, systemic chemotherapy is not recommended for the treatment of neuroendocrine tumors of non-pancreatic origin. Exceptions to this include the treatment of grade 2 neuroendocrine tumors, aggressive tumors demonstrating RECIST progression within 3–6 months of the initiation of other therapies, or those tumors which are somatostatin receptor-negative. In this setting, the metronomic administration of temozolomide +/− capecitabine is an accepted therapeutic option, or capecitabine/bevacizumab as a last-line therapy [44,45,46].

In contrast to well- to moderately differentiated neuroendocrine tumors, cisplatin-based cytotoxic chemotherapy (cisplatin/etoposide) is the standard first-line therapy in the treatment of poorly differentiated, grade 3 neuroendocrine tumors of any origin. Although remission rates as high as 67% have been reported in these cases, the median progression-free survival is meager at 4–6 months [47,48]. Second-line therapy for grade 3 neuroendocrine tumors includes FOLFOX and FOLFIRI [49,50] in most cases with temozolomide-based regimens being preferentially used in grade 3 pancreatic neuroendocrine tumor or gastroenteropancreatic neuroendocrine tumor of other primary site with Ki-67 < 55% [48,51].

### 3.5. Peptide Receptor Radionuclide Therapy

Peptide receptor radionuclide therapy may be used as second-line therapy in patients with progressive, homogenously somatostatin receptor-positive neuroendocrine tumors. Treatment is performed by the administration of ^90^Y- or ^177^Lu-labeled somatostatin analogs. Specific eligibility requirements as outlined by ENETS include homogenous disease uptake on somatostatin receptor imaging with tumor uptake being at least as high as normal liver uptake. Additionally, patients must present with inoperable disease, have a life expectancy of at least 3–6 months, and an ECOG score <4 [52]. While traditionally ^90^Y-labeled somatostatin analogs are used for this modality of treatment, increasingly, ^177^Lu has found favor given a lower propensity for renal toxicity. The NETTER-1 trial, which examined the use of ^177^Lu-DOTATATE in the treatment of progressive midgut neuroendocrine tumor, demonstrated significantly increased progression-free survival compared to high-dose octreotide alone. Given the results of this trial in conjunction with other prospective and retrospective studies, peptide receptor radionuclide therapy is generally recommended as second-line therapy discussed above [53,54,55]. The COMPOSE trial is an ongoing phase III multicenter, randomized controlled trial comparing the use of ^177^Lu-edotreotide versus everolimus in grade 1 and 2 metastatic gastroenteropancreatic neuroendocrine tumors. The primary endpoint is progression-free survival with a secondary endpoint of overall survival to be reported [56]. The COMPETE trial represents another ongoing phase III multicenter, randomized controlled trial evaluating the use of ^177^Lu-edotreotide in patients with inoperable, progressive somatostatin receptor-positive gastroenteropancreatic neuroendocrine tumors compared to everolimus. The primary endpoint is progression-free survival with secondary endpoints including objective response rate, median duration of disease control, overall survival, and quality of life measures [57].

## 4. Conclusions

GEP-NET with PSM is poorly understood based on the current body of literature. Acceptable management algorithms may be devised by the extrapolation of data from the studies of other patient populations, but there is a need for studies directed at patients with GEP-NET presenting with PSM specifically. Though randomized studies may be impossible to perform given the rarity of this disease process, retrospective and prospective studies should be performed with attention to overall survival in the setting of different systemic therapy regimens. Additionally, additional studies should be designed to elicit the optimal patient populations to whom to offer cytoreductive surgery to improve survival, and additional investigation into the role of hyperthermic intraperitoneal chemotherapy with regard to overall survival should be performed.

## Figures and Tables

**Table 1:** Summary of studies evaluating use of heated intraperitoneal chemotherapy in peritoneal metastasis of gastroenteropancreatic neuroendocrine tumor.

Author	Year	Patients (*n*):	HIPEC Agents	HIPEC Duration and Temperature	Survival Data
Elias et al. [18]	2014	41	IV leucovorin/5-FU Intraperitoneal oxaliplatin or oxaliplatin + Irinotecan at differing concentrations - The number of each not defined	Time: 30 min Temperature: 43 °C	5-year overall survival: 69%
Brandl et al. [19]	2017	14	Not defined	Time: 90 min for 83.3% of patients Temperature: Variable (mean 40.9 °C)	Survival at 16 month follow-up: 43%
Goere et al. [20]	2017	127	Cisplatin, doxorubicin, mitomycin C, oxaliplatin, or irinotecan - The number of patients undergoing each not defined	Time: 25–180 min Temperature: Variable (mean 41.9 °C)	5-year overall survival: 39.9%
Hajjar et al. [21]	2022	67	Not defined	Not defined	5-year overall survival: 91.6% (CRS alone); 74.5% (CRS + HIPEC)
